# Pharmacological characterization of a high-affinity *p*-tyramine transporter in rat brain synaptosomes

**DOI:** 10.1038/srep38006

**Published:** 2016-11-30

**Authors:** Mark D. Berry, Shannon Hart, Anthony R. Pryor, Samantha Hunter, Danielle Gardiner

**Affiliations:** 1 Department of Biochemistry, Memorial University of Newfoundland, St. John’s, NL, A1B 3X9, Canada

## Abstract

*p*-Tyramine is an archetypal member of the endogenous family of monoamines known as trace amines, and is one of the endogenous agonists for trace amine-associated receptor (TAAR)1. While much work has focused on the function of TAAR1, very little is known about the regulation of the endogenous agonists. We have previously reported that *p*-tyramine readily crosses lipid bilayers and that its release from synaptosomes is non-exocytotic. Such release, however, showed characteristics of modification by one or more transporters. Here we provide the first characterization of such a transporter. Using frontal cortical and striatal synaptosomes we show that *p*-tyramine passage across synaptosome membranes is not modified by selective inhibition of either the dopamine, noradrenaline or 5-HT transporters. In contrast, inhibition of uptake-2 transporters significantly slowed *p*-tyramine re-uptake. Using inhibitors of varying selectivity, we identify Organic Cation Transporter 2 (OCT2; *SLC22A2*) as mediating high affinity uptake of *p*-tyramine at physiologically relevant concentrations. Further, we confirm the presence of OCT2 protein in synaptosomes. These results provide the first identification of a high affinity neuronal transporter for *p*-tyramine, and also confirm the recently described localization of OCT2 in pre-synaptic terminals.

Trace amines are a family of endogenous amines synthesized in neurones and found in all species examined^1^. Archetypal members of this family include *p*-tyramine, 2-phenylethylamine, and tryptamine. Although they are present in very low levels, indeed the term was initially intended to represent any endogenous amine with a tissue concentration below 100 ng/g tissue^2^, they are heterogeneously distributed throughout the brain^1^. In 2001, a family of vertebrate G protein-coupled receptors, subsequently termed trace amine-associated receptors (TAAR), was identified, a sub-set of which were selectively activated by the trace amines^3^,^4^. Subsequently much effort has been devoted to the study of these receptors, in particular TAAR1, which has been shown to modulate dopaminergic^5^,^6^,^7^,^8^,^9^ serotonergic^6^ and glutamatergic^6^,^10^,^11^ transmission; interact with dopamine (DAT)^12^,^13^,^14^, noradrenaline (NET)^14^, 5-HT (SERT)^14^ and glutamate (EAAT2)^15^ transporters; decrease craving for various psychostimulants^16^,^17^,^18^; and regulate appetite^10^,^19^, sleep^7^,^10^ and cognitive function^7^,^10^. Based on this, TAAR1 agonists and/or partial agonists have been proposed as novel therapeutics for schizophrenia^10^ and drug abuse^20^.

While impressive advances have occurred with respect to determining TAAR1 pharmacology and physiology, there is still very little known about the homeostatic processes in place to control the endogenous agonists. The archetypal trace amines are synthesized by decarboxylation of the pre-cursor amino acids l-tyrosine, l-phenylalanine and l-tryptophan via the enzyme aromatic l-amino acid decarboxylase (AADC; EC 4.1.1.28)^1^. Degradation occurs primarily via monoamine oxidase (MAO; EC 1.4.3.4)-A and -B^1^, with 2-phenylethylamine still the only known endogenous compound showing high selectivity for MAO-B^21^. While this metabolic pathway is analogous to those of the monoamine neurotransmitters, trace amines have a remarkable turn-over rate, the half-life for the endogenous pool being less than 30 seconds^22^. Such a high turn-over suggests that trace amines are not stored, consistent with previous reports of a lack of vesicular storage^23^. Consistent with this we have shown that both *p*-tyramine and 2-phenylethylamine readily diffuse across synthetic lipid bilayers^24^. Further, previous research indicated that neither *p*-tyramine nor 2-phenylethylamine release from neuronal preparations was increased by potassium-induced depolarization^24^,^25^,^26^, indicating that trace amine release does not occur by exocytosis, consistent with simple diffusion across the lipid bilayer. Under such a situation synaptic levels of trace amines would be in a steady state, controlled solely by the relative rates of synthesis and degradation.

The lack of increase of trace amine release following depolarization, not only indicates a non-exocytotic release, but also suggests that one or more transporters are involved in regulating synaptic levels. At physiological pH trace amines, being primary amines, will carry a net positive charge. As such the intracellular:extracellular equilibrium ratio can be predicted at any membrane potential from the Nernst equation. As shown in Fig. 1, under such situations the predicted concentration ratio for a species carrying a single positive charge, and that freely diffuses across membranes, varies from a greater than 10:1 intracellular preference at typical resting membrane potentials, to an approximate 2:1 extracellular preference at full depolarization. As such, the absence of an increased release of *p*-tyramine following depolarization clearly indicates the presence of other factors that prevent this change in distribution. We reasoned that this most likely represents the presence of a transporter that re-uptakes released *p*-tyramine into the nerve terminal.

A number of known transporter proteins have been reported to include trace amines in their substrate profile, although this has rarely been examined at physiologically-relevant, nanomolar levels. Such transporters can be broadly classified as neuronal and extraneuronal, definitions which generally correspond to the uptake-1 and uptake-2 concept originally proposed by Iverson^27^. While recent studies have suggested that this is an over-simplification^28^,^29^, for ease of discussion we will refer here to uptake-1 and uptake-2 transporters.

Uptake-1 transporters are typified by DAT, NET and SERT, all members of the Slc6 family, and show high selectivity, but low capacity, Na-dependent transport, that are generally viewed as being primarily responsible for synaptic clearance of released neurotransmitters^30^. Trace amines have long been recognized as being substrates for these transporters^31^,^32^,^33^. This, however, has only been demonstrated at high micromolar, or even millimolar, levels, at least three orders of magnitude in excess of the maximum synaptic concentration (approximately 100 nM) thought to be possible for trace amines under normal conditions^1^. As such, it is unlikely that these transporters contribute to the synaptic clearance of trace amines unless some other, previously undescribed, regulatory event is occurring.

With respect to neuronal monoamines, uptake-2 transporters are typified by the Organic Cation Transporter (OCT; Slc22A1-3) family of transporters and Plasma Membrane Monoamine Transporter (PMAT; Slc29A4). Classically these are thought of as polyspecific, low-selectivity, high capacity transporters that mediate overflow clearance of synaptic neurotransmitters when uptake-1 transporters become saturated^34^. Although far less studied, all four of these transporters have been reported to include one or more trace amines in their substrate profile^35^,^36^,^37^,^38^,^39^, with at least one, OCT1, showing nanomolar affinity for *p*-tyramine^35^,^40^. OCT1, however, is not thought to be present in neurones^41^,^42^ making it unlikely to play a role in the effects previously observed. In contrast, OCT2^41^,^43^,^44^, OCT3^42^,^45^ and PMAT^46^,^47^ have all been reported to be expressed in neurones.

The aim of the current study was to systematically examine the effect of selective inhibition of individual transporters on the release characteristics of *p*-tyramine from pre-loaded frontal cortical and striatal synaptosome preparations, and characterize the kinetics of any transporter-mediated passage observed. Although selective inhibitors of uptake-1 transporters are well characterized, there are few available selective inhibitors of the various uptake-2 transporters. We therefore adopted a subtractive, deductive approach to assess the role of uptake-2 transporters in controlling *p*-tyramine extra-neuronal levels following inhibition of various transporter combinations.

## Results

### Effect of depolarization on *p*-tyramine release

Initial studies confirmed that a 10 minute incubation at 37 °C with 100 nM [^3^H]*p*-tyramine was sufficient to give equilibration of loading in both cortical and striatal synaptosomes (data not shown). High potassium-induced depolarization of synaptosomes prepared from either frontal cortex (Fig. 2a; F = 0.8929 (3, 314), *P* = 0.4451, n = 20) or striatum (Fig. 2b; F = 1.574 (3, 362), *P* = 0.1953, n = 23) did not increase release of pre-loaded *p*-tyramine. Although there was some evidence of a plateau region (t = 2–5 min) in the release curves, a two-phase decay function did not unambiguously fit the data, and therefore a one-phase exponential model was accepted.

### Effect of Uptake-1 inhibitors on *p*-tyramine release

Selective inhibition of DAT (Fig. 3a; F = 0.3480 (3, 42), *P* = 0.7908, n = 3), NET (Fig. 3b; F = 1.063 (3, 58), *P* = 0.3718, n = 4) or SERT (Fig. 3c; F = 0.1259 (3, 42), *P* = 0.9442, n = 3) was without effect on *p*-tyramine release characteristics from either cortical (Fig. 3) or striatal synaptosomes, under either basal (Fig. 3) or depolarizing (see Supplemental Fig. S1) conditions.

### Effect of Uptake-2 inhibitors on *p*-tyramine release

The pan OCT + PMAT inhibitor, decynium-22^34^,^37^, significantly increased apparent release of *p*-tyramine under both basal (Fig. 4a; F = 5.261 (3, 58), *P* = 0.0028, n = 4) and depolarizing (Fig. 4b; F = 4.473 (3, 58), *P* = 0.0068, n = 4) conditions. This was primarily manifest as a decrease in the half-life of the release curve under both conditions (Basal = 3.01 min, Basal + decynium-22 = 1.24 min; Depolarizing = 3.07 min, Depolarizing + decynium-22 = 0.77 min). Similar effects were observed in both cortical and striatal preparations.

The somewhat more selective inhibitor quinidine (pan OCT inhibition, no PMAT inhibition)^48^,^49^ gave similar effects to decynium-22, increasing the apparent *p*-tyramine release under both basal (Fig. 5a; F = 106.6 (3, 58), *P* < 0.0001, n = 4) and depolarizing (Fig. 5b; F = 72.07 (3, 58), *P* < 0.0001, n = 4) conditions. Again, responses were associated with a pronounced decrease in the half-life for *p*-tyramine release (Basal = 2.52 min., Basal + quinidine = 0.82 min.; Depolarizing = 3.10 min., Depolarizing + quinidine = 0.91 min.). The selective PMAT inhibitor lopinavir^50^ was completely devoid of effects under either basal (Fig. 5c; F = 0.645 (3, 58), *P* = 0.5895, n = 4) or depolarizing (Fig. 5d; F = 0.094 (3, 58), *P* = 0.9630, n = 4) conditions. Again, essentially identical responses to each inhibitor were obtained in striatal and cortical preparations.

Selective inhibition of OCT3 with corticosterone^36^,^51^ failed to alter *p*-tyramine release characteristics under either basal (Fig. 6a; F = 0.078 (3, 58), *P* = 0.9716, n = 4) or depolarizing (Fig. 6b; F = 0.021 (3, 58), *P* = 0.9957, n = 4) conditions in either brain region. Pentamidine, an OCT1 + OCT2 inhibitor^52^, however, significantly increased the apparent rate of release of *p*-tyramine under basal (Fig. 7a; F = 3.008 (3, 42), *P* = 0.041, n = 3) and depolarizing (Fig. 7b; F = 2.930 (3, 42), P = 0.045, n = 3) conditions. As before, this was primarily due to a pronounced decrease in the release half-life (Basal = 3.44 min., Basal + pentamidine = 1.21 min.; Depolarizing = 2.26 min., Depolarizing + pentamidine = 0.52 min.). In contrast, the selective OCT1 inhibitor atropine^48^ was without effect under basal (Fig. 7c; F = 0.311 (3, 42), *P* = 0.8175, n = 3) and depolarizing (Fig. 7d; F = 0.2394 (3, 42), *P* = 0.8683, n = 3) conditions. The same response profile to inhibitors was observed in both striatal (Fig. 7) and cortical (see Supplemental Fig. S2) preparations.

The above studies suggest that *p*-tyramine is transported across synaptosomal membranes by OCT2, or at least a transporter with an OCT2-like pharmacological profile. To further validate OCT2 as the transporter, and rule out the possibility that effects were due to the inhibition of multiple transporters removing non-selective redundancy, we repeated studies with a cocktail of selective inhibitors that would inhibit OCT1 (atropine), OCT3 (corticosterone) and PMAT (lopinavir), but leave OCT2-mediated transport intact. This cocktail of inhibitors was without effect on *p*-tyramine release characteristics under both basal (F = 0.5212 (3, 58), *P* = 0.6694, n = 4) and depolarizing (F = 0.2931 (3, 58), *P* = 0.8302, n = 4) conditions (see Supplemental Fig. S3). Further, Western blot analysis confirmed the presence of OCT2 in the synaptosomal preparations (Fig. 8 main panel).

We next characterized the kinetics of the transporter. The ready diffusion of *p*-tyramine across lipid bilayers in the absence of membrane transporters is a potential confound to such studies. We therefore defined total and diffusion-mediated uptake as that occurring in the absence and presence of 400 μM pentamidine respectively, with the difference between the two giving transporter mediated uptake (Fig. 9). Using this approach, Michaelis-Menten analysis gave a pentamidine-sensitive uptake with a V_max_ = 30.2 fmol/mg protein/s and a K_t_ = 101.5 nM.

## Discussion

The identification of a family of vertebrate G protein-coupled receptors, at least some of which are selectively activated by 2-phenylethylamine and *p*-tyramine^3^,^4^, has catalyzed a resurgence of interest in the so-called trace amines. While this has led to notable advances in elucidating the pharmacology and physiology of TAAR1, there is still a relative lack of understanding about the homeostatic mechanisms in place to regulate the endogenous ligands. The activity of the synthetic enzyme AADC is known to be regulated in response to dopaminergic^53^,^54^,^55^ and noradrenergic^56^ receptor activation, an effect that alters the rate of synthesis of 2-phenylethylamine^57^, but not dopamine^58^,^59^. Metabolism occurs primarily via MAO, with 2-phenylethylamine preferentially metabolized by MAO-B^21^, and *p*-tyramine a mixed MAO-A/MAO-B substrate^22^,^60^.

**Figure 1:**
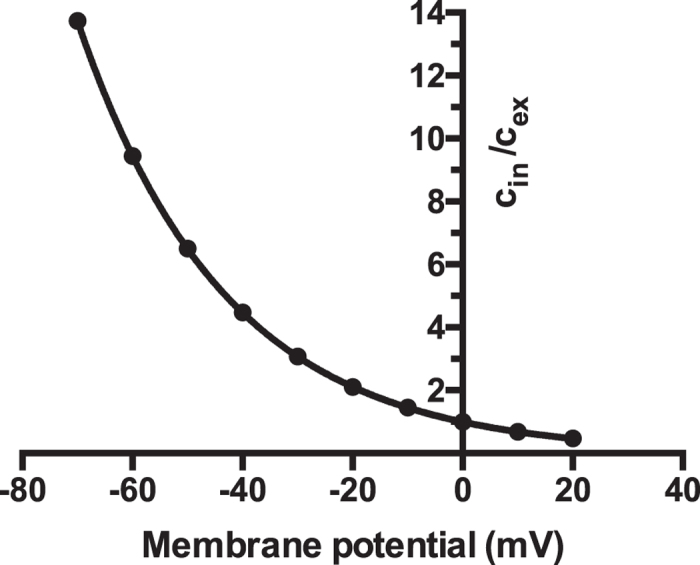
Predicted ratio of intracellular: extracellular concentrations for a singly charged cation at various membrane potentials. The intracellular:extracellular concentration ratio were predicted from the Nernst equation. c_in_ = intracellular concentration, c_ex_ = extracellular concentration.

**Figure 2:**
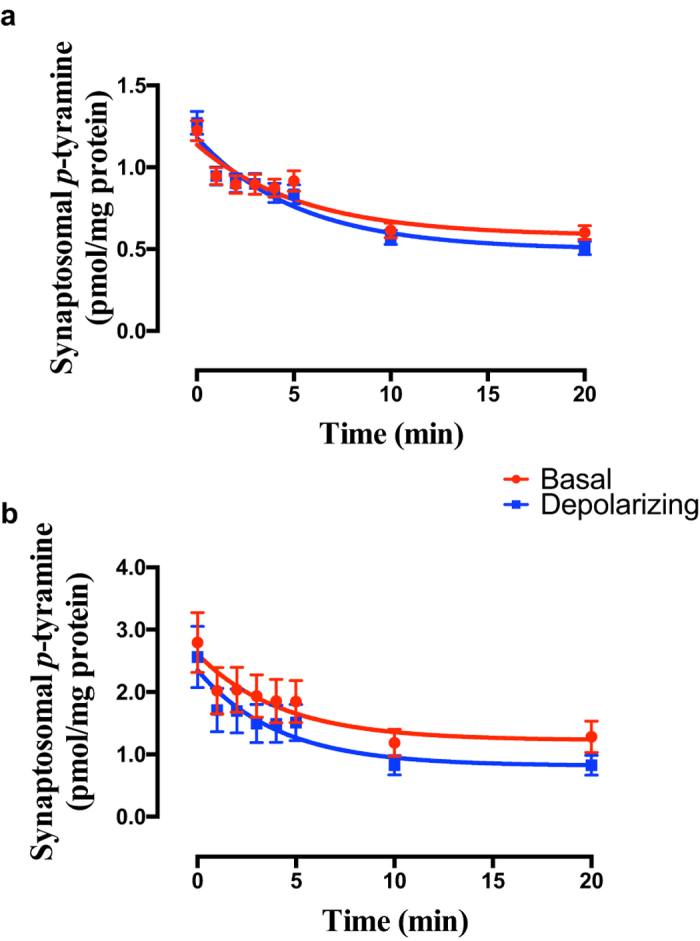
The effect of membrane depolarization on release of *p*-tyramine from (**a**) frontal cortex and (**b**) striatal synaptosome preparations. Synaptosomes were pre-loaded by incubation with 100 nM [^3^H]*p*-tyramine × 10 min. Depolarization was induced by 25 mM KCl. Release curves were fit to a one-phase exponential decay function and compared by Extra sum-of-squares F-test. Data represents mean ± sem, of 20 (frontal cortex) and 23 (striatum) independent experiments.

Previous studies have suggested that neither 2-phenylethylamine nor *p*-tyramine are stored in synaptic vesicles; their extracellular levels are solely determined by whole tissue levels^26^, and their release from nerve terminals is not increased by depolarization^25^,^26^. On this basis it was previously suggested that the trace amines readily diffuse across neuronal membranes, with their synaptic levels being in a steady-state, controlled by the relative rates of synthesis and degradation^1^,^61^. There is some evidence to support free diffusion of trace amines across biological membranes^62^,^63^, although others have suggested the involvement of an unknown transporter^64^,^65^. We previously provided the first direct measure of trace amine diffusion across lipid bilayers devoid of proteins, and confirmed that this occurred at a significantly faster rate than that of the monoamine neurotransmitters dopamine, noradrenaline and 5-HT^24^. Further, we confirmed the earlier observations of Dyck^26^, showing that neither 2-phenylethylamine nor *p*-tyramine were released from synaptosomes in an activity-dependent manner^24^. Together the results suggest that not only does trace amine release occur through a non-exocytotic process, but that a re-uptake protein may be present in nerve terminals. As primary amines both 2-phenylethylamine and *p*-tyramine are positively charged at physiological pH. As such their passage across a lipid bilayer can be predicted from the Nernst equation. From this it can be clearly seen that in the absence of other processes, diffusion-mediated release should increase in response to membrane depolarization (see Fig. 1): that this does not occur (Fig. 2) is most easily explained by the presence of a transporter which re-uptakes released *p*-tyramine. We have previously confirmed that dopamine release from the same preparations is increased on high-potassium induced depolarization^24^, verifying functionality of our synaptosome preparations. The current study provides the first pharmacological characterization of a high affinity *p*-tyramine transporter.

**Figure 3:**
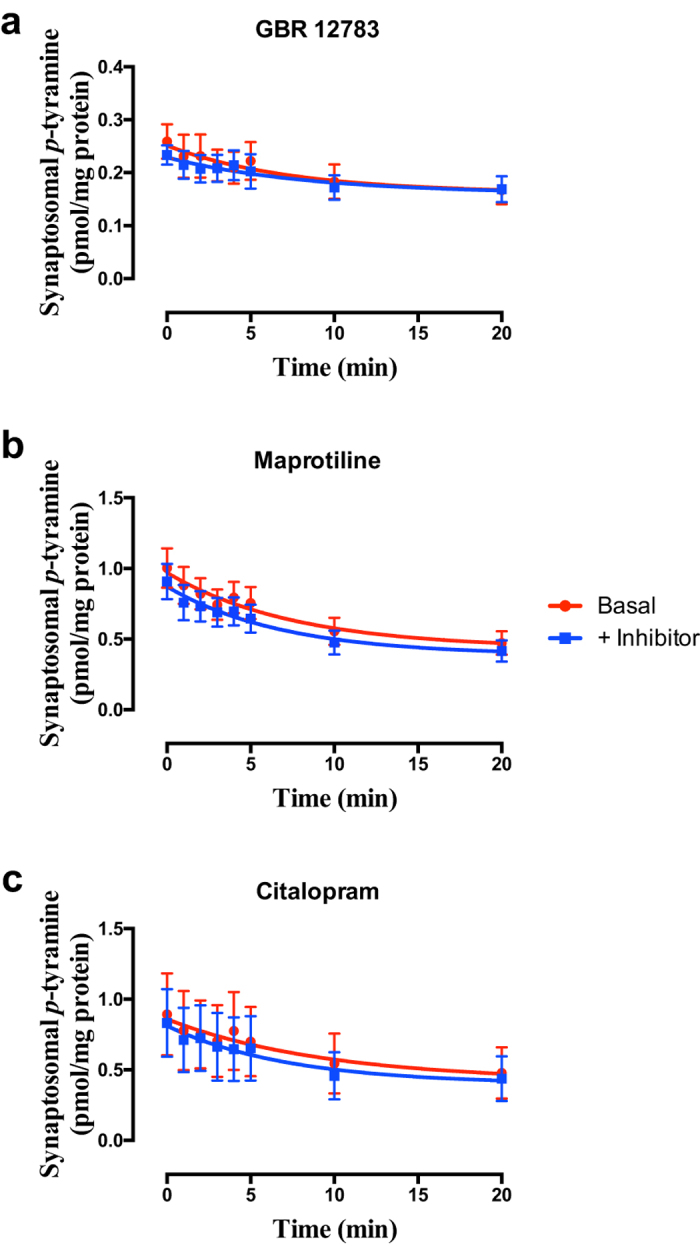
Selective inhibition of (a) DAT, (b) NET or (c) SERT does not alter *p*-tyramine release characteristics. Synaptosomes prepared from frontal cortex were pre-loaded by pre-incubation with 100 nM *p*-tyramine as previously described. Release under basal (non-depolarizing) conditions was measured in the absence and presence of 50 nM GBR 12783 (**a**), 100 nM maprotiline (**b**), or 50 nM citalopram (**c**), curves fit to a one-phase exponential decay function. Curves obtained in the absence and presence of individual inhibitors were compared by Extra sum-of-squares F-test. Data represents mean ± sem, n = 3 (GBR 12783, citalopram), 4 (maprotiline).

A number of known transporters have been reported to include one or more trace amines in their substrate profile, although this has almost exclusively been reported only at supra-physiological concentrations. The endogenous levels of trace amines are in the low ng/g tissue range, which is estimated to correspond to approximately 10–100 nM tissue levels^1^. Here we pre-loaded synaptosome preparations by incubation with 100 nM [^3^H]*p*-tyramine to approximate the physiological concentrations at which uptake is expected to occur. Consistent with previous studies indicating *p*-tyramine is only transported at millimolar concentrations^31^,^32^,^33^, selective inhibition of either DAT (Slc6A3), NET (Slc6A2) or SERT (Slc6A4), was without effect on *p*-tyramine release characteristics (Fig. 3), confirming that under physiological conditions *p*-tyramine is not a substrate for these transporters.

**Figure 4:**
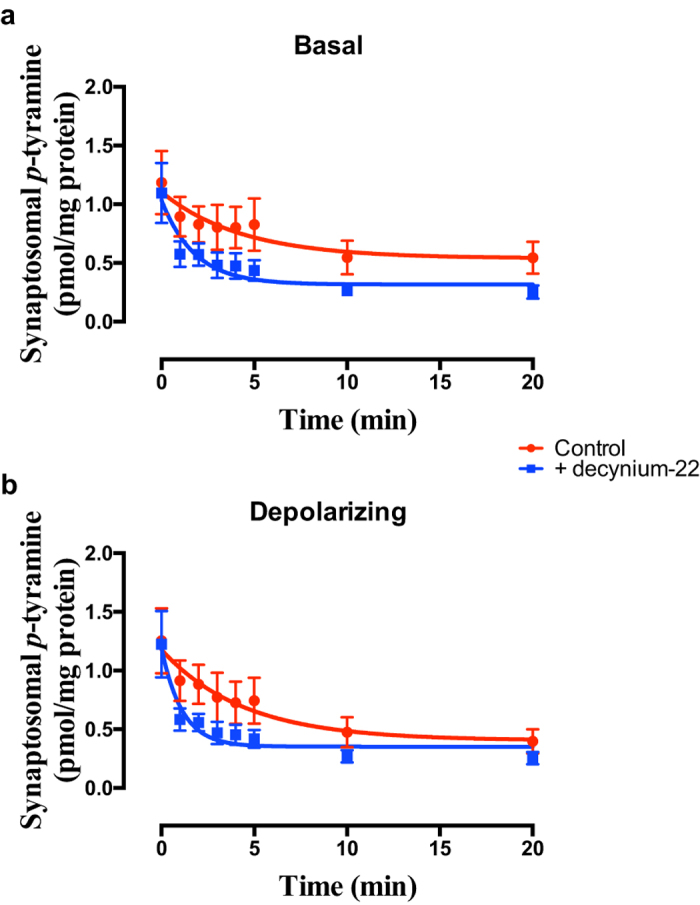
Decynium-22 increases the apparent rate of release of p-tyramine from synaptosome preparations under (a) basal and (b) depolarizing conditions. Frontal cortical synaptosome preparations were pre-loaded with [^3^H]*p*-tyramine as previously described, and release in the absence and presence of 1 μM decynium-22 determined under basal (**a**) and depolarizing (**b**) conditions. Depolarization was induced by incubation in the presence of 25 mM KCl. Release curves were fit to a one-phase exponential decay function and compared in the absence and presence of decynium-22 by Extra sum-of-squares F-test in each condition. Data represents mean ± sem of 4 independent experiments.

Unlike the Slc6 family of transporters, occasional reports have suggested that *p*-tyramine is a substrate for one or more of the poly-specific OCT (Slc22A1-3) and PMAT (Slc29A4) transporters, and this may occur at concentrations as low as 100 nM^35^,^40^. Since there are few truly selective inhibitors of individual members of the Slc22/Slc29 families described we investigated the effects of inhibition of various combinations of the transporters on *p*-tyramine release characteristics in an effort to identify the potential transporter. Decynium-22, a non-selective inhibitor of OCT1-3 and PMAT^34^,^37^, significantly increased the apparent rate of release of *p*-tyramine (Fig. 4). Although OCT transporters have been reported to be bi-directional^66^, and as such increased release via the transporter in the presence of decynium-22 is possible, the most likely explanation is that re-uptake of *p*-tyramine was being inhibited.

**Figure 5:**
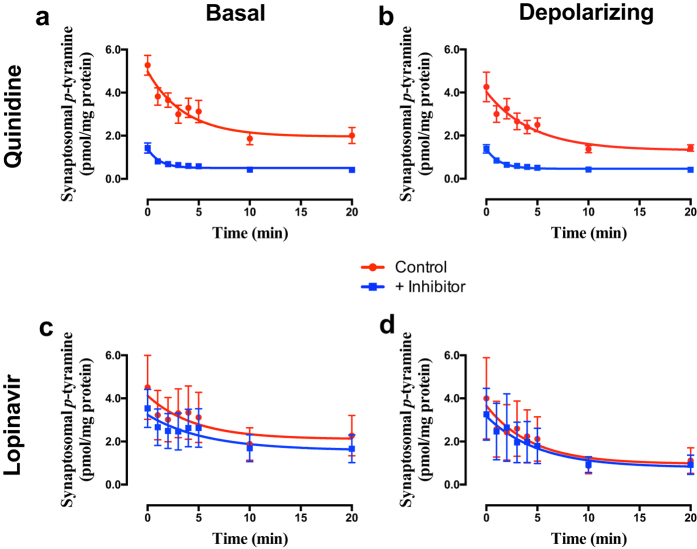
The effects of quinidine (a,b) and lopinavir (c,d) on *p*-tyramine release from synaptosomes under basal (a,c) and depolarizing (b,d) conditions. Pre-loaded synaptosomes prepared from striatum were incubated under basal or depolarizing conditions in the absence and presence of either 50 μM quinidine or 30 μM lopinavir. Depolarization was induced by incubation in the presence of 25 mM KCl. Release curves under each condition were fit to a one-phase exponential decay function and curves obtained in the absence or presence of inhibitor compared by Extra sum-of-squares F-test in each condition. Data represents mean ± sem of 4 independent experiments.

**Figure 6:**
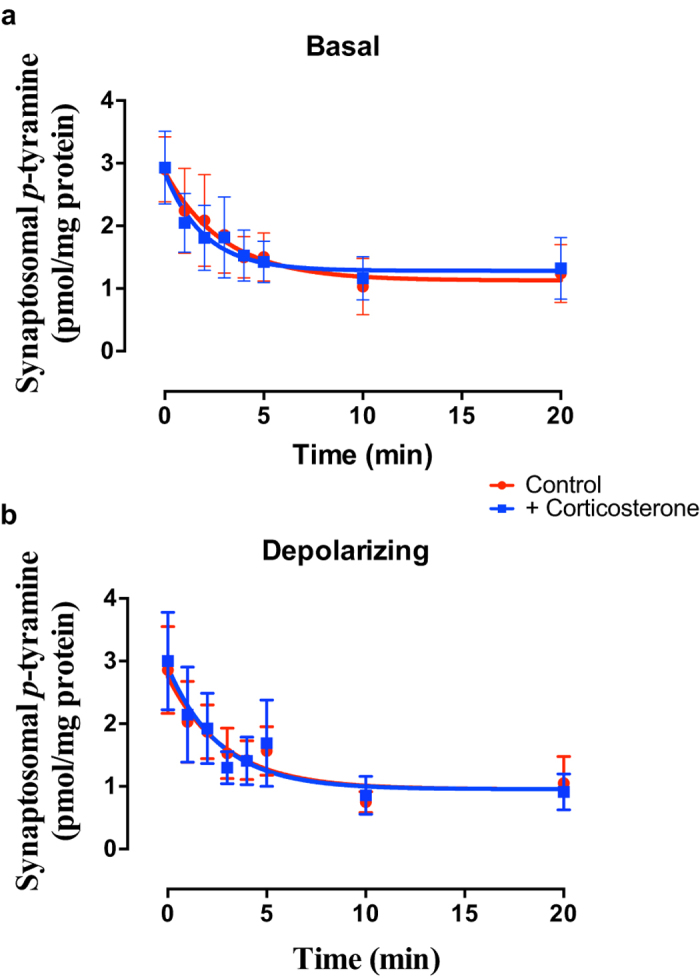
Corticosterone does not alter *p*-tyramine release characteristics under either (a) basal or (b) depolarizing conditions. Pre-loaded striatal synaptosomes were incubated under basal (5 mM KCl) or depolarizing (25 mM KCl) conditions in the absence and presence of 1 μM corticosterone. Release curves under each condition were fit to a one-phase exponential decay function and curves obtained in the absence or presence of inhibitor compared by Extra sum-of-squares F-test in each condition. Data represents mean ± sem, n = 4.

**Figure 7:**
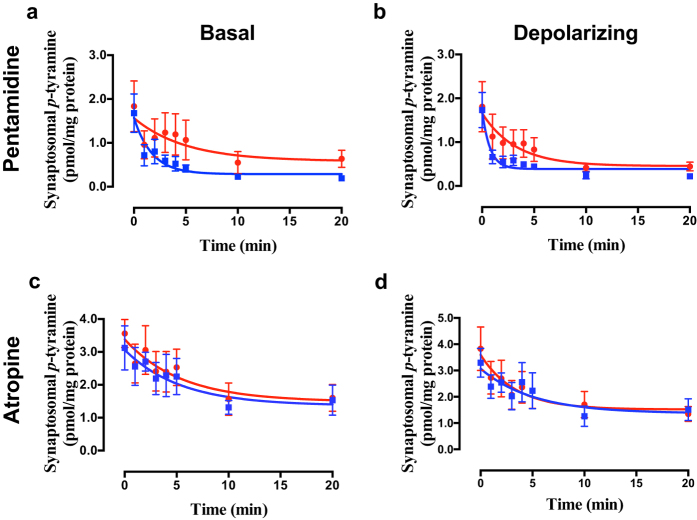
The effects of pentamidine (a,b) and atropine (c,d) on *p*-tyramine release from synaptosomes under basal (a,c) and depolarizing (b,d) conditions. Pre-loaded striatal synaptosomes were incubated under basal (5 mM KCl) or depolarizing (25 mM KCl) conditions in the absence and presence of either 200 μM pentamidine or 10 μM atropine. Release curves under each condition were fit to a one-phase exponential decay function and curves obtained in the absence or presence of inhibitor compared by Extra sum-of-squares F-test in each condition. Data represents mean ± sem of 3 independent experiments.

Quinidine, an inhibitor of OCT1-3, but not PMAT^48^,^49^, had similar effects to decynium-22 (Fig. 5a,b), suggesting that PMAT may not be involved in transporting *p*-tyramine. This was further confirmed by the lack of effect of the PMAT-selective inhibitor, lopinavir^50^ (Fig. 5c,d). Corticosterone, a selective inhibitor of OCT3^36^,^51^, was also without effect (Fig. 6), suggesting that either OCT1 or OCT2 was responsible for *p*-tyramine transport. This was further confirmed by a pentamidine-induced increase in apparent rate of *p*-tyramine release (Fig. 7a,b). Since OCT1 is reported to not be present in central neurones^41^,^42^, we reasoned that OCT2 (or a transporter sharing its pharmacological profile) was acting as a transporter of nanomolar concentrations of *p*-tyramine in rat brain synaptosomes. A lack of OCT1-mediated transport was further confirmed by the absence of an effect of atropine (Fig. 7c,d), a selective inhibitor of OCT1^48^.

Since the above results had only shown effects with compounds that inhibited more than one transporter, we further confirmed selective transport by an OCT2-like transporter by examining whether a cocktail of selective inhibitors altered *p*-tyramine release characteristics. The combination of atropine, corticosterone, and lopinavir, to inhibit OCT1, OCT3 and PMAT but leave OCT2 activity intact, was also without effect on release characteristics (see Supplemental Fig. S2). This indicated that *p*-tyramine release characteristics were not being modified as a consequence of inhibiting multiple transporters and thus removing transport redundancy, but that transport across neuronal membranes was mediated solely by OCT2, or an as yet unknown transporter sharing a pharmacological profile with OCT2. There are a number of members of the Slc22 family that are currently poorly characterized, including various organic cation transporter-like proteins^67^, and at this moment we cannot definitively exclude these from playing a role in the effects observed here. Selective knock-down and/or transfection of OCT2 in cell lines would help to address this, although isolated synaptosomes are not readily amenable to such an approach.

**Figure 8:**
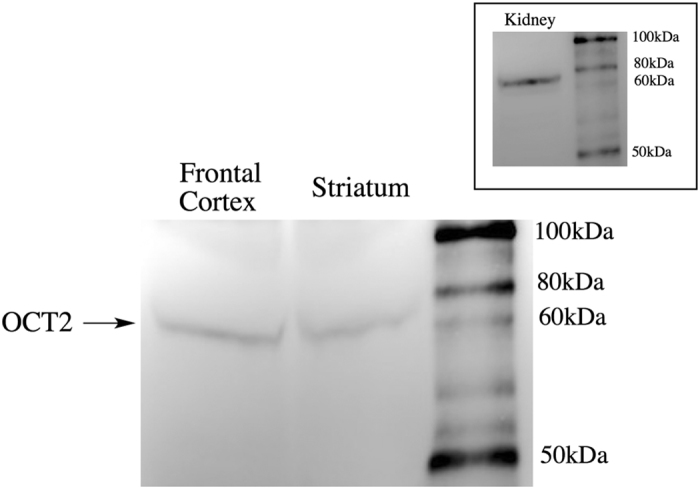
OCT2 is present in frontal cortical and striatal synaptosome preparations. Main panel: Proteins from synaptosome preparations (200 μg total protein) of frontal cortex and striatum were separated on a denaturing 4–12% gradient bis-tris acrylamide gel prior to transfer to a nitrocellulose membrane. Anti-SLC22A2 antibody staining identified a protein with a molecular mass of approximately 66 kDa, consistent with OCT2. Inset: The same band was identified in kidney homogenates. Images are representative blots. Identical results were obtained in each of three independent experiments.

**Figure 9:**
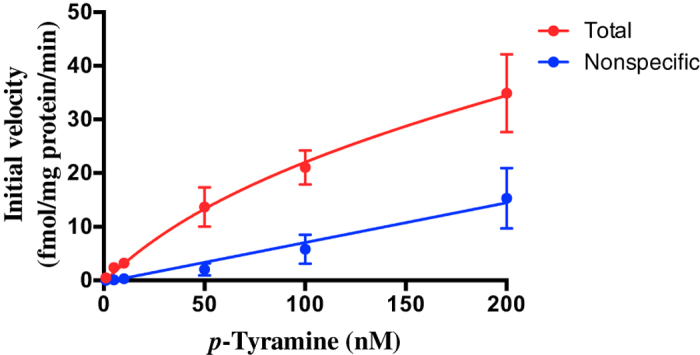
Michaelis-Menten plots of [^3^H]*p*-tyramine uptake in the presence and absence of pentamidine. Frontal cortex synaptosomes were incubated in the presence of increasing concentrations of *p*-tyramine and uptake terminated at various time points. From the resulting curves the initial rate of uptake was determined at each concentration. Total uptake is defined as that occurring in the absence of 400 μM pentamidine. Non-specific uptake is defined as that occurring in the presence of pentamidine. The pentamidine-sensitive uptake is then defined from the difference between the two curves. Values represent mean ± sem of 4 independent experiments.

We confirmed the presence of OCT2 in both frontal cortex and striatal synaptosome preparations by Western blot (Fig. 8). A predominant band at approximately 66 kDa, corresponding to the predicted molecular mass of rat OCT2^68^, was observed in both striatal and frontal cortical synaptosomes. This is consistent with the recent report of OCT2 presence in monoaminergic and cholinergic pre-synaptic terminals^69^, including cholinergic synaptic vesicles^70^. OCT2 is predominantly expressed in the kidney^66^ and as a positive control we confirmed the same sized band was also present in whole kidney homogenates (c.f. Fig. 8 main panel and inset).

Finally, we sought to determine the kinetics of *p*-tyramine transport by the pentamidine-sensitive transporter. The ready diffusion of *p*-tyramine across lipid bilayers (t_½_ ≈ 15 s)^24^ is a complicating factor in this regard. We therefore defined total (transporter-+diffusion-mediated) and pentamidine-insensitive (diffusion-mediated) uptake by synaptosomes with the difference equating to the pentamidine-sensitive transporter-mediated uptake. Using such an approach Michaelis-Menten analysis revealed kinetic parameters for uptake of k_t_ = 101.5 nM and V_max_ = 30.2 fmol/mg protein/s. The k_t_ is in good agreement with estimates of the endogenous tissue concentrations of *p*-tyramine, indicating that uptake by this transporter is likely to be physiologically relevant. The V_max_ equates to a value of 1.8 pmol/mg protein/min and is similar to values reported for the selective transport of dopamine by DAT^71^ or serotonin by SERT^72^ in synaptosome preparations. Although we controlled for diffusion of *p*-tyramine into the synaptosomes, we cannot prevent diffusion back out, and this may well result in the apparent net V_max_ obtained being an underestimate of the true value.

It is interesting to note that the receptor target of *p*-tyramine, TAAR1, is not only predominantly located intracellularly^4^,^73^, necessitating membrane passage for post-synaptic mediated effects, but is also rather broadly tuned in terms of ligand selectivity^74^,^75^. The OCT family as a whole, including OCT2, also has a very broad substrate selectivity^76^, although few compounds have been reported to show the nanomolar affinity that we observed for *p*-tyramine. Both d-amphetamine and MDMA (3,4-methylenedioxymethamphetamine) are reported to show a greater affinity for OCT2 than other family members^42^ and both are also high affinity ligands for TAAR1^4^. As such, there are similarities between the substrate selectivity of OCT2 and the ligand selectivity of TAAR1. Whether other trace amines such as 2-phenylethylamine are also transported by OCT2 requires further study. 2-Phenylethylamine membrane passage was, however, previously reported to occur independently of all known OCT^65^.

It is also interesting to note that OCT2 knock-out animals were recently reported to show deficits in GSK-3β signalling^44^, and TAAR1 activation has been shown to recruit the β-arrestin-2/GSK-3β transduction cascade^9^,^73^. TAAR1 is well established to be either constitutively active or tonically activated by endogenous ligands^5^,^7^, and this raises the possibility that the deficits observed in OCT2 knock-out animals may be, at least in part, due to a decrease in tonic TAAR1 activation due to decreased membrane passage of *p*-tyramine in the absence of OCT2. In such a situation one would predict that TAAR1 agonists such as RO5166017^77^ may reverse the phenotype associated with OCT2 knock-out. Finally, a high affinity transport of *p*-tyramine by OCT2 also suggests that alterations in trace amine homeostasis should be considered with respect to the “off target” pharmacological profiles of the diverse therapeutics^78^,^79^ that are known to interact with OCT2.

In conclusion, the current study provides the first pharmacological characterization of a high affinity *p*-tyramine transporter present in rat brain synaptosomal preparations. This transporter has a profile consistent with OCT2, which we confirm to be present in synaptosomes from both frontal cortex and striatum. This also provides further support for a pre-synaptic terminal location of OCT2. The kinetics of the uptake of *p*-tyramine by this transporter are consistent with those shown for the uptake of the monoamine neurotransmitters by their selective transporters. This study enhances the understanding of the normal homeostatic mechanisms involved in the control of trace aminergic functioning in the vertebrate central nervous system.

## Methods

### Animals

All studies were conducted in accordance with Canadian Council on Animal Care guidelines and were approved by the Memorial University of Newfoundland institutional animal care committee. Male Wistar rats (200–400 g) were used in all studies, and were housed two per cage at 20 ± 1 °C, 40–70% humidity, on a 12 hour light:dark cycle with food and water *ad libitum*. All procedures were conducted during the light phase of the cycle.

### Synaptosome Preparation

Synaptosomes were prepared as previously described^24^ from the pooled tissue of 4 (kinetics) or 5 (release studies) animals for each independent experiment which determined [^3^H]*p*-tyramine release under both basal and depolarizing conditions in the absence and presence of one or more transporter inhibitor(s). Briefly, pooled frontal cortices or striata were homogenized in 10 volumes of 0.32 M ice-cold sucrose and the resultant suspension centrifuged at 4 °C at 1,000 g × 10 min. The supernatant was removed and further centrifuged at 4 °C at 10,000 g × 20 min. The resultant supernatant was discarded and the P2 pellet re-suspended in ice-cold assay buffer (25 mM HEPES, 120 mM NaCl, 5 mM KCl, 2.5 mM CaCl_2_, 1.2 mM MgSO_4_, 2 μg/mL d-glucose, 0.2 μg/mL ascorbic acid; pH 7.5; 1 mL buffer per pair of frontal cortices or striata). All buffers also included 10 μM pargyline, 100 μM diethyldithiocarbamate and 2.5 μM OR-486 to prevent metabolism of *p*-tyramine. Samples of all synaptosome preparations were stored frozen at −20 °C for subsequent protein assay and/or Western blot analysis.

### Release Assays

Synaptosomes (50 μL aliquots) were pre-loaded by incubation with 100 nM [^3^H]*p*-tyramine (0.1 Ci/mmol; American Radiolabeled Chemicals Inc., St. Louis, MO) at 37 °C × 10 min. Preliminary studies confirmed that this was sufficient for uptake of the added *p*-tyramine to have reached equilibrium. Uptake was stopped by the addition of 1 mL ice-cold assay buffer and immersion of the reaction tube in ice, followed by centrifugation at 10,000 g × 4 min at 4 °C. The supernatant was discarded and the pellet washed by re-suspension in 0.5 mL ice-cold assay buffer and re-centrifugation. For studies in which the effects of transporter inhibitors were examined, this buffer was supplemented with inhibitor(s) at the indicated concentration(s), and transporter inhibitors included in all subsequent buffer solutions. The resultant pellets were re-suspended in either basal or depolarizing (25 mM KCl) assay buffer. The following inhibitors were used: GBR-12783 (50 nM; DAT inhibition), citalopram (50 nM; SERT inhibition^80^), maprotiline (100 nM, NET inhibition), decynium-22 (1 μM; pan-OCT + PMAT inhibition), lopinavir (30 μM; PMAT inhibition), quinidine (50 μM; pan-OCT inhibition^81^), atropine (10 μM; OCT1 inhibition), corticosterone (1 μM; OCT3 inhibition), and pentamidine (200 μM; OCT1 and OCT2 inhibition). The selectivity of the various inhibitors used in this study are summarized in Supplemental Table 1^80^,^81^,^82^.

Pre-loaded synaptosomes were incubated at 37 °C for varying times. Immediately following incubation samples were centrifuged at 10,000 g × 4 min at 4 °C. The supernatants were removed, the pellets re-suspended in 0.5 mL NP-40 lysis buffer (20 mM Tris-HCl, 137 mM NaCl, 10% (v/v) glycerol, 1% (v/v) NP-40, 2 mM EDTA, pH 8.0) and incubated at 37 °C × 30 min. Following incubation suspensions were transferred to individual scintillation vials and 5 mL scintillation cocktail (ScintiSafe, ThermoFisher Scientific, Ottawa, ON) added and allowed to stand overnight. Total tritium in each sample was then counted by liquid scintillation counting using a Tri-Carb 2810TR liquid scintillation counter (Perkin Elmer, Waltham, MA) operating at 60–65% efficiency. Total dpm in each sample was converted to pmol *p*-tyramine by comparison to a standard curve prepared with each experiment. In each experiment the average of duplicate samples at each time point and treatment was used for data analysis. Data was normalized to the total protein content of the synaptosomal preparation and fit to a one-phase exponential decay function using GraphPad Prism 6.0 (GraphPad, LaJolla, CA).

### Transporter kinetics

Synaptosome aliquots were incubated at 37 °C with [^3^H]*p*-tyramine at varying concentrations for various time points in basal assay buffer in the absence and presence of 400 μM pentamidine. *p*-Tyramine uptake was stopped by the addition of 1 mL of ice-cold assay buffer and immediately placing the reaction vessel in an ice-bath, followed by centrifugation at 4 °C at 10,000 g × 4 min. The supernatant was discarded and the pellet re-suspended in 0.5 mL NP-40 lysis buffer and counted for total tritium as described above. Total dpm in each sample were converted to pmol *p*-tyramine by comparison to standard curves prepared with each experiment. All samples were assayed in duplicate with the average of the two readings used for data analysis. Values were normalized to the total protein content of synaptosomal preparations.

Plots of total *p*-tyramine uptake versus time were constructed and the initial (<45 s) linear rate of uptake (v_o_) for each *p*-tyramine concentration determined by linear regression. Michaelis-Menten plots of v_o_ versus *p*-tyramine concentration in the absence and presence of 400 μM pentamidine were then used to determine the k_t_ and v_max_ of pentamidine-sensitive uptake using GraphPad Prism 6.0.

### Protein Assay

Total protein in synaptosomal preparations was determined with a Pierce bicinchoninic acid (BCA) kit (ThermoFisher Scientific, Ottawa, ON) as per manufacturer’s instructions. Briefly, samples were diluted with 0.9% saline (frontal cortex 1:20; striatum 1:10) and 10 μL of the diluted sample incubated with BCA reagent at 37 °C × 30 min. Absorbance was determined at 562 nm using a BioTek Synergy 2 microplate reader (BioTek, Winooski, VT), and total protein determined by comparison to a bovine serum albumin standard curve prepared with each assay.

### Western Blot

Synaptosome preparations were diluted with basal HEPES buffer to a protein concentration of 50 μg/mL. Proteins were separated on pre-made Bolt^®^ 4–12% bis-tris acrylamide gels (ThermoFisher Scientific, Ottawa, ON) under denaturing conditions, and then transferred to a nitrocellulose membrane using an iBlot 2 dry blotting system (ThermoFisher Scientific, Ottawa, ON) following pre-soaking in 20% ethanol x 5 min as per the manufacturer’s instructions. Western blot was performed using the iBind Western system (ThermoFisher Scientific, Ottawa, ON) using a rabbit polyclonal anti-SLC22A2 antibody (eLabScience, Bethesda, MD) at a dilution of 1:200, and horseradish peroxidase conjugated goat anti-rabbit secondary antibody (Bio-Rad, Mississauga, ON) at a dilution of 1:600. Following blotting, the membrane was washed with deionized water and chemiluminescence induced by incubating with the Clarity Western ECL substrate (50:50 (v/v) hydrogen peroxide: luminol/enhancer solution; Bio-Rad, Mississauga, ON) × 5 min. Chemiluminescence was visualized using an ImageQuant LAS4000 gel documentation system (GE Healthcare Life Sciences, Mississauga, ON). Bands were compared to a biotinylated protein ladder kit (Cell Signaling Technology, Whitby, ON) visualized by chemiluminescence following incubation with the provided horseradish peroxidase-conjugated anti-biotin antibody.

### Data Analysis and Statistics

All synaptosome release and kinetic studies were analyzed using global curve fit functions in GraphPad Prism 6.0. Release curves were fit to a one-phase exponential decay function and compared by Extra sum-of-squares F-test, with α = 0.05, and significance taken at *P* < 0.05.

## Additional Information

**How to cite this article**: Berry, M. D. *et al*. Pharmacological characterization of a high-affinity *p*-tyramine transporter in rat brain synaptosomes. *Sci. Rep.* **6**, 38006; doi: 10.1038/srep38006 (2016).

**Publisher's note:** Springer Nature remains neutral with regard to jurisdictional claims in published maps and institutional affiliations.

## Supplementary Material

Supplementary Information
