# Supplementary material for: Pharmacological characterization of a high-affinity p-tyramine transporter in rat brain synaptosomes
Source: Sci Rep. 2016 Nov 30;6:38006. doi: 10.1038/srep38006 (PMC5128819; doi:10.1038/srep38006)
Supplement: Supplementary Information [file srep38006-s1.pdf]

Pharmacological characterization of a high-affinity *p*-tyramine transporter in rat brain synaptosomes

Mark D. Berry*, Shannon Hart, Anthony R. Pryor, Samantha Hunter and Danielle Gardiner

Supplementary Information

Supplemental Table 1 Representative IC_{50} and/or Ki values of transporter inhibitors

	OCT1	OCT2	OCT3	PMAT	DAT	NET	SERT
**Decynium-22**	1-5 μM[39,51]	0.1-1 μM[39,51]	0.1 μM[51]	0.1 μM[37]			
**Quinidine**	5-18 μM[39]	7-87 μM[39]	14-124 μM[39]				
**Pentamidine**	0.4-16 μM[52]	5-11 μM[52]					
**Corticosterone**	7-22 μM[39,51]	5-34 μM [39,51]	0.1-0.3 μM[39,51]	450 μM[37]			
**Atropine**	1.2 μM[48]	29 μM[48]	466 μM[48]				
**Lopinavir**	174 μM[50]	No effect[50]	No effect[50]	1.4 μM[50]			
**GBR 12783**					2 nM[80]	95 nM[80]	1 μM[80]
**Maprotiline**						11 nM[81]	6 μM[81]
**Citalopram**					9 μM[82]	6 μM[82]	2 nM[82]

In the current study, compounds were used at 10-20x the reported IC_{50} and/or Ki values in order to produce in excess of 90% inhibition of the transporter of interest.

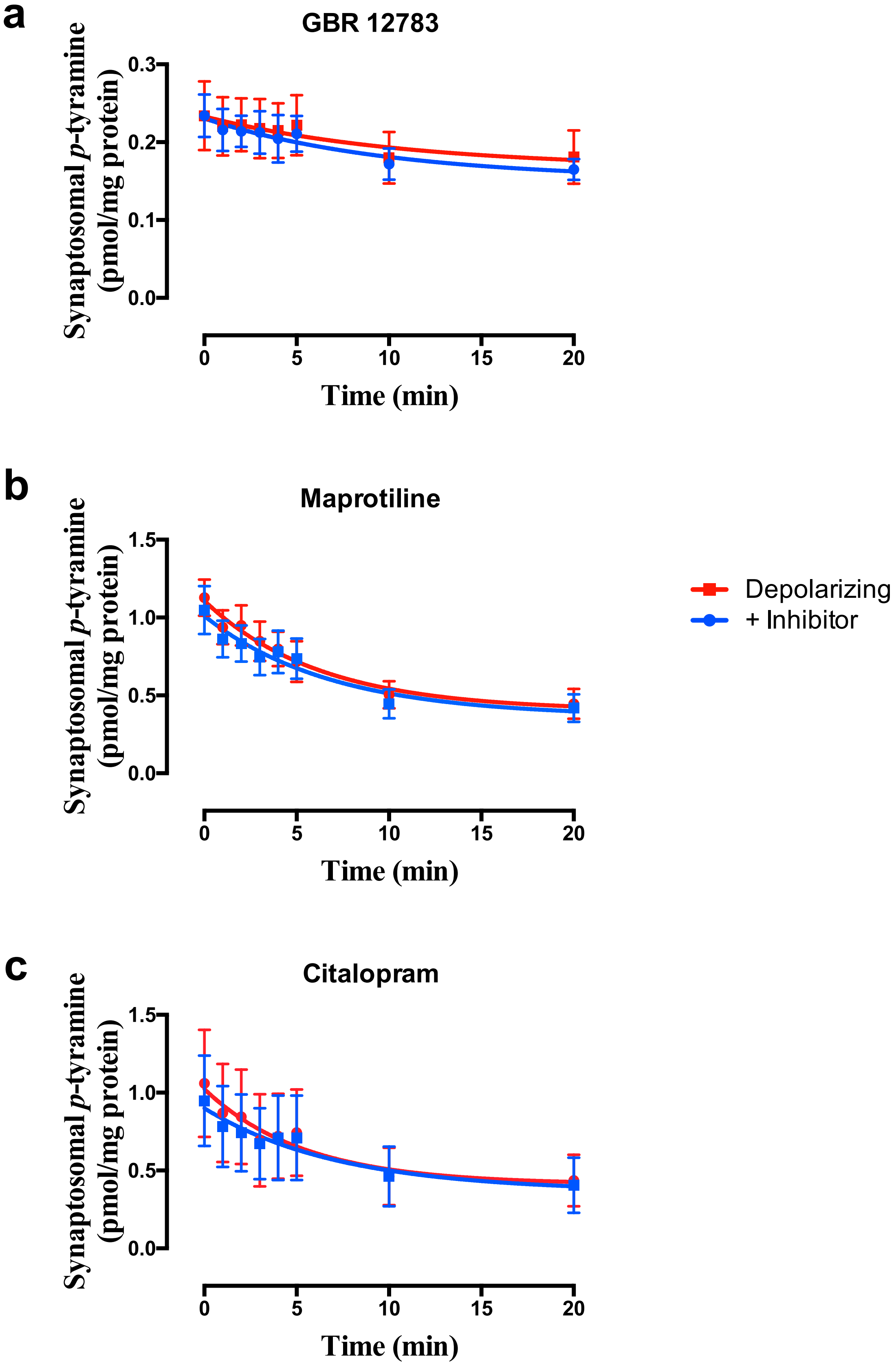

a
GBR 12783
Synaptosomal p-tyramine (pmol/mg protein)
0.0
0.1
0.2
0.3
0
5
10
15
20
Time (min)
b
Maprotiline
Synaptosomal p-tyramine (pmol/mg protein)
0.0
0.5
1.0
1.5
0
5
10
15
20
Time (min)
Depolarizing
+ Inhibitor
c
Citalopram
Synaptosomal p-tyramine (pmol/mg protein)
0.0
0.5
1.0
1.5
0
5
10
15
20
Time (min)

Fig. S1. Selective inhibition of (A) DAT, (B) NET or (C) SERT does not alter *p*-tyramine release characteristics under depolarizing conditions. Synaptosomes prepared from frontal cortex were pre-loaded by incubation with 100 nM *p*-tyramine as previously described. Release under depolarizing (25 mM KCl) conditions was measured in the absence and presence of 50 nM GBR 12783 (A), 100 nM maprotiline (B), or 50 nM citalopram (C) and curves fit to a one-phase exponential decay function. Curves obtained in the absence and presence of individual inhibitors were compared by Extra sum-of-squares F-test. Data represents mean ± s.e.m. from 3 (GBR 12783, citalopram) or 4 (maprotiline) independent experiments.

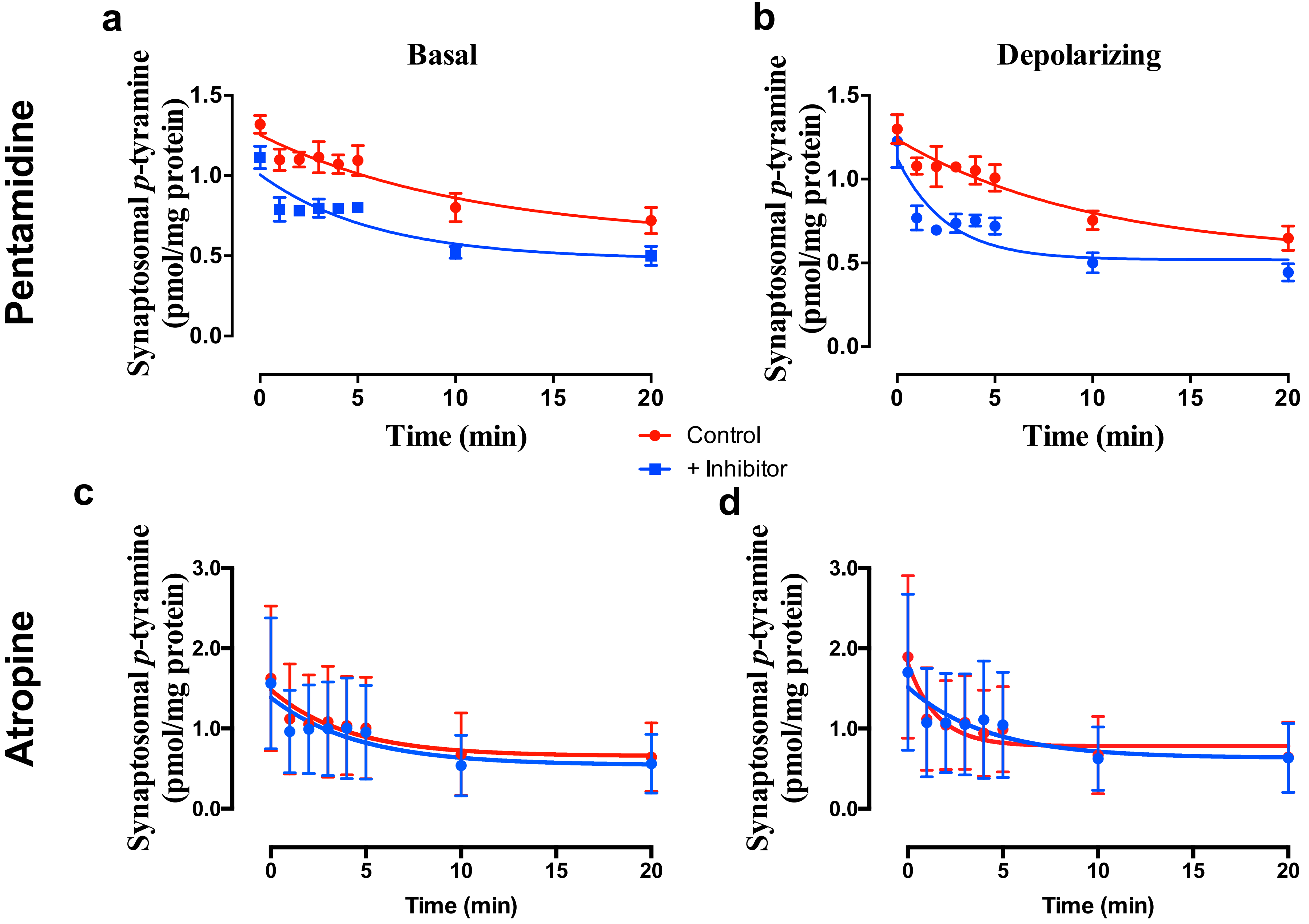

a
b
Pentamidine
Basal
Depolarizing
Synaptosomal p-tyramine (pmol/mg protein)
Time (min)
Control
+ Inhibitor
c
d
Atropine
Synaptosomal p-tyramine (pmol/mg protein)
Time (min)

Fig. S2. The effects of pentamidine (A, B) and atropine (C, D) on *p*-tyramine release from synaptosomes under basal (A, C) and depolarizing (B, D) conditions. Pre-loaded frontal cortex synaptosomes were incubated under basal (5 mM KCl) or depolarizing (25 mM KCl) conditions in the absence and presence of either 200 μM pentamidine or 10 μM atropine. Release curves under each condition were fit to a one-phase exponential decay function and curves obtained in the absence or presence of inhibitor compared by Extra sum-of-squares F-test in each condition. Pentamidine basal $F = 21.14$ (3,42) $P < 0.0001$; depolarization $F = 15.67$ (3,42) $P < 0.0001$. Data represents mean ± s.e.m. of 3 (pentamidine) or 2 (atropine) independent experiments.

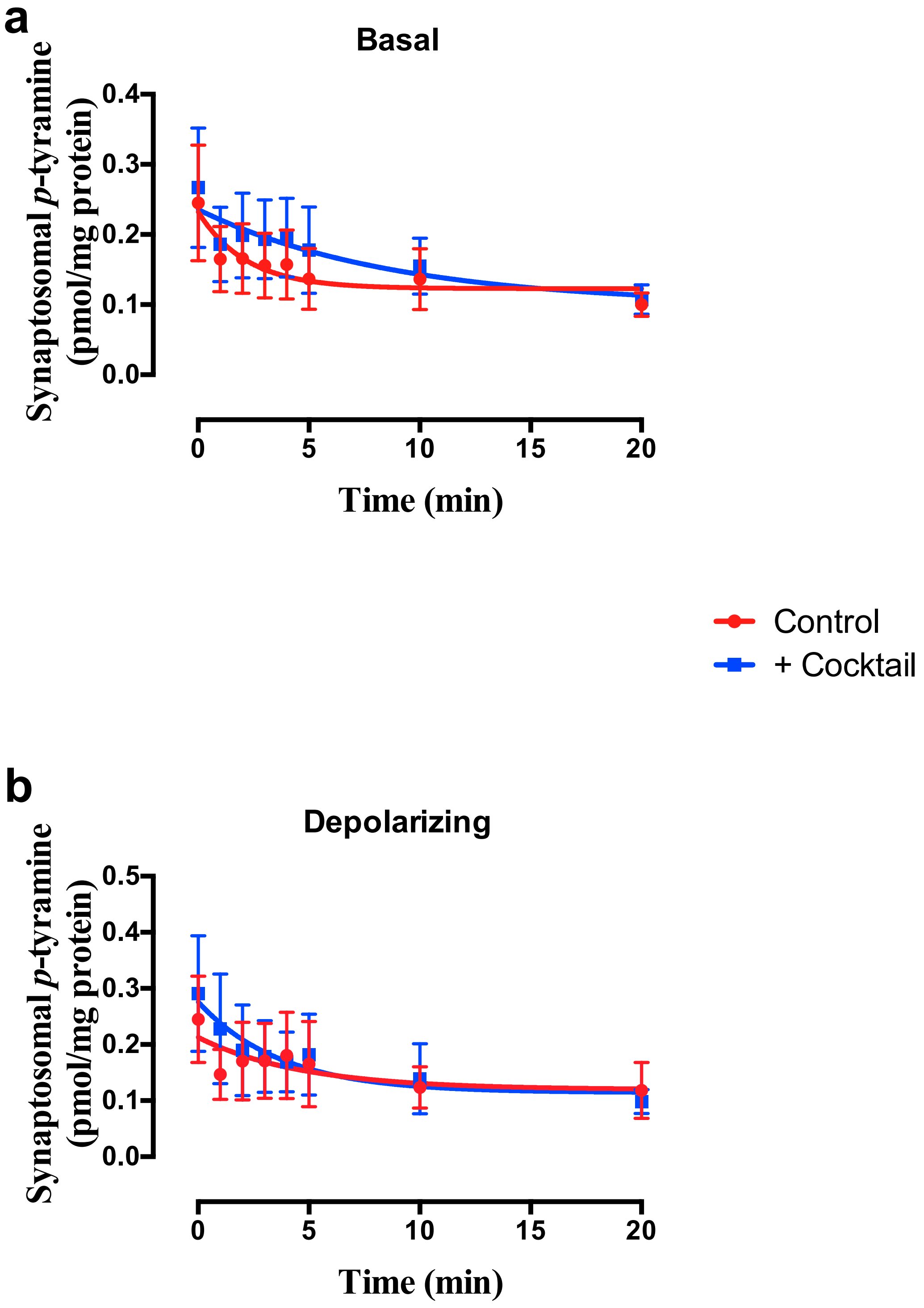
a
Basal
Synaptosomal p-tyramine (pmol/mg protein)
0.4
0.3
0.2
0.1
0.0
0
5
10
15
20
Time (min)
Control
+ Cocktail
b
Depolarizing
Synaptosomal p-tyramine (pmol/mg protein)
0.5
0.4
0.3
0.2
0.1
0.0
0
5
10
15
20
Time (min)

Fig. S3. Combined inhibition of OCT1, OCT3 and PMAT does not affect *p*-tyramine release characteristics under (A) basal or (B) depolarizing conditions. Synaptosomes prepared from frontal cortex were pre-loaded with [^{3}H]*p*-tyramine as previously described and subsequent release determined in the presence and absence of a cocktail of inhibitors. OCT1 was inhibited with 10 μM atropine, OCT3 with 1 μM corticosterone, and PMAT with 30 μM lopinavir. Depolarization was induced with 25 mM KCl. Release curves under each condition were fit to a one-phase exponential decay function and curves obtained in the absence or presence of the inhibitor cocktail compared by Extra sum-of-squares F-test under basal and depolarizing conditions. Data represents mean ± s.e.m., n=4.
